# Influence of Dietary Fiber and Polyphenols During Pre-Gestation, Gestation, or Lactation on Intestinal Gene Expression

**DOI:** 10.3390/nu17020341

**Published:** 2025-01-18

**Authors:** Daniela Ceballos-Sánchez, Laura Sáez-Fuertes, Sergi Casanova-Crespo, Maria J. Rodríguez-Lagunas, Margarida Castell, Francisco J. Pérez-Cano, Malen Massot-Cladera

**Affiliations:** 1 Physiology Section, Department of Biochemistry and Physiology, Faculty of Pharmacy and Food Science, University of Barcelona (UB), 08028 Barcelona, Spain; daniceballoss@ub.edu (D.C.-S.); laurasaezfuertes@ub.edu (L.S.-F.); sergi.casanova@ub.edu (S.C.-C.); mjrodriguez@ub.edu (M.J.R.-L.); margaridacastell@ub.edu (M.C.); malen.massot@ub.edu (M.M.-C.); 2 Nutrition and Food Safety Research Institute (INSA-UB), 08921 Santa Coloma de Gramenet, Spain; 3 Centro de Investigación Biomédica en Red de Fisiopatología de la Obesidad y la Nutrición (CIBEROBN), Instituto de Salud Carlos III, 28029 Madrid, Spain

**Keywords:** Mediterranean diet, maternal diet, fiber, polyphenols, gut, gene expression, small intestine

## Abstract

Background/Objectives: Diet composition is important for health, especially during critical periods such as pre-gestation (P), gestation (G), or lactation (S), due to its potential impact not only on the mother but on the offspring. The Mediterranean diet includes many healthy foods rich in fiber and/or polyphenols, such as whole grains, fruits, vegetables, beans, and nuts. The present preclinical study assesses the impact of a diet rich in fiber and polyphenols (HFP diet) during one of those three periods (P, G, or S, three weeks each) on the rat gene expression of the small intestine obtained at the end of the lactation period. Methods: This analysis was performed by the mRNA two step PCR amplification by random primers and poly-T, followed by library generation and HiSeq X-Ten Illumina sequencing (Seqplexing), and further confirmed by Real time PCR and ELISA. Results: The results showed a broad number of genes significantly modulated after the HFP diet compared to the reference diet, with a higher number of genes modulated when the supplementing period was closer to the analysis day (S > G > P). Notably, genes involved in immune signaling, intestinal absorption, and cell growth were among those more significantly affected by the HFP dietary intervention. The HFP diet influenced the expression of key genes such as ferritin, fatty acid synthase, apelin, and complement proteins, among others. There was a unique gene modified in all the intervention periods (Family with Sequence Similarity 117 Member A, *Fam117A*, which codifies a protein with unknown function), indicating that this molecule may participate critically in the effects induced by fiber and polyphenols during these periods. Conclusions: Overall, in rats, the influence of diet for a three-week period around birth is able to modulate the intestinal gene expression, and consequently, maternal health, which can eventually have an indirect impact on the offspring.

## 1. Introduction

The term “exposome” describes all environmental or non-genetic factors to which humans are exposed throughout the course of their lives [1]. Among these factors, diet is particularly important for health, especially during critical periods such as pre-gestation, gestation, or lactation, due to its potential impact on both the mother and the offspring. However, the molecular mechanisms induced by the diet and its impact on birth outcomes and long-term infant health are still unclear [2].

Dietary components such as fiber and polyphenols, as a part of a healthy or Mediterranean diet, are two of the most extensively studied bioactive compounds [3,4]. Recent research highlights significant effects on various physiological and pathological conditions, including metabolic regulation, gut health, and inflammation. Dietary fiber contributes to improved glycemic control, cardiovascular health, and digestive function [4,5,6]. Meanwhile, polyphenols, including flavonoids, phenolic acids, and lignans, exhibit potent antioxidant and anti-inflammatory properties, among others. Both bioactive compounds interact synergistically with gut microbiota, increasing the production of metabolites such as short-chain fatty acids (SCFAs) and phenolic derivatives, which exert local and systemic effects [4,6,7].

Emerging evidence emphasizes the importance of the maternal diet during pregnancy and lactation in shaping maternal and fetal/infant health outcomes. A maternal diet rich in fiber has been shown to modulate gut microbiota composition, enhance intestinal barrier function, and influence metabolic homeostasis [8,9]. Similarly, polyphenols, often bound to dietary fiber, enhance its bioactive properties, and may contribute to anti-inflammatory and antioxidant mechanisms [8,9,10]. These effects are mediated, in part, through modifications in gene expression, particularly in the intestinal epithelium, where nutrient signaling pathways and transcriptional networks are activated [10]. In this regard, dietary fiber and polyphenols influence processes such as DNA methylation, histone modification, and non-coding RNA regulation, which collectively alter chromatin accessibility and the transcription of specific genes [11,12]. In addition, SCFAs and polyphenols interact with key enzymes, such as DNA methyltransferases (DNMTs) and histone deacetylases (HDACs), to regulate the transcriptional landscape of various tissues, including the intestine [13,14,15]. Such modifications are particularly significant during critical developmental periods, such as gestation and lactation, when maternal diet can shape the epigenetic and gene expression profiles in both mothers and their offspring.

Taking all the previous literature into account, we can hypothesize that a diet high in fiber and polyphenols given to mothers during pre-gestation, gestation, or lactation, could impact their intestinal gene expression. To test this, we aimed to determine, at a preclinical level, how a maternal diet enriched in fiber and polyphenols during these periods influence the gene expression in the small intestine. Our findings may help uncover the molecular pathways influenced by these dietary compounds and provide insights into how maternal nutrition can serve as a tool for improving both maternal and infant health outcomes.

## 2. Materials and Methods

### 2.1. Animals

Six-week-old female Wistar rats (*n* = 40) were obtained from Janvier Labs (Saint-Berthevin, France) and housed in the experimental animal facility in the Diagonal-Campus of the Faculty of Pharmacy and Food Science (University of Barcelona, UB). After a one-week acclimatization period, the females entered the pre-gestation period, which was established in our experimental design in 21 days. They were then placed in the males’ cages (1/2 proportion for four days). Following mating, the females were separated from the males and housed individually.

The animals were kept under controlled environmental conditions, which included humidity (50–55%), temperature (21 ± 2 °C), and carefully controlled 12-h light–dark cycles. Animals had ad libitum access to food and water. The animal procedures followed were approved by the Ethics Committee for Animal Experimentation of the University of Barcelona (CEEA/UB ref. 240/19) and the Generalitat de Catalunya (DAAM 10933). The necessary sample size was determined using the Appraising Project Office’s program from the Universidad Miguel Hernández de Elche (Alicante, Spain). This calculation, based on intestinal immunoglobulin A (IgA) and *Toll Like* Receptor 4 (TLR4) gene expression as the variables, assumed no dropout rate and a two-sided type I error of 0.05. In addition, the sample size was adjusted following the University Ethical Committee guidelines and the three Rs rule for animal experiments was applied.

### 2.2. Diets and Experimental Design

Two diets were used: a standard diet based on the AIN-93G diet (Envigo, Indianapolis, IN, USA) and an experimental diet (HFP diet) that was formulated considering the fiber and polyphenol content typical of a healthy dietary profile observed in a gestating/lactating Spanish Mediterranean cohort [16]. The HFP diet was based on the AIN-93G formula, enriched with 8% inulin (Fibruline™, from Chicory roots, Cosucra, Warcoing, Belgium) and 1% pectin (PE21006 from citrus fruits, Gojira Fine Chemicals, LLC, Mundelein, IL, USA) as fiber, and a 0.5% polyphenol mixture, primarily consisting of flavonoids (Table 1). The added flavonoids were all purchased from Millipore Sigma (Madison, WI, USA) as follows: Catechin (C1251), Epicatechin (E1753), Hesperidin (H5254), Naringenin (N5893), and Quercetin (Q4951). This diet was supplied by Envigo, vacuum-packed to prevent oxidation and contamination, and stored at 4 °C until used.

The animals were randomly assigned to four groups (*n* = 6 pregnant rats/group). Groups were based on the diet the animals received and the feeding period: a control group (REF group), which received the standard diet throughout the study (9 weeks), and three experimental groups that received the experimental diet (HFP) during a specific life stage (each one lasting three weeks): the pre-gestation group (P group), which were fed the HFP diet only for three weeks before mating, the gestation group (G group), which received HFP only during gestation (three weeks in rats), and the lactation group (S group), fed the HFP diet only during the lactation period (three weeks in rats). At the end of the lactation period, small intestine samples were obtained from all groups.

### 2.3. Sample Collection and Preparation

At the end of the nine-week experimental period, the rats were intramuscularly anesthetized with ketamine (90 mg/kg) (Merial Laboratorios, S.A., Barcelona, Spain) and xylazine (10 mg/kg) (Bayer A.G., Leverkusen, Germany). A 5 mm segment from the middle section of the small intestine was aseptically collected from all animals and preserved in RNAlater^®^ at 4 °C overnight. Blood samples were collected from the heart into tubes containing ethylenediaminetetraacetic acid (EDTA) for the quantification of ferritin and adiponectin. The tissue samples were stored at −20 °C for subsequent gene expression analysis using microarrays/Real Time PCR and ELISA quantification, respectively.

### 2.4. RNA Extraction

Tissue samples were placed into lysing matrix tubes (MP Biomedicals, Illkirch, France) containing a suitable buffer and homogenized using a FastPrep^®^ instrument (MP Biomedicals) for 30 s. The resulting lysates were centrifuged at 12,000× *g* for 3 min to remove residual tissue debris. RNA was then extracted using the RNeasy Mini Kit (Qiagen, Madrid, Spain) according to the manufacturer’s protocol and quantified using a NanoDrop spectrophotometer with the NanoDrop IVD-1000 v.3.1.2 software (NanoDrop Technologies, Wilmington, DE, USA). To analyze the results, Agilent 2100 Bioanalyzer with the RNA 6000 LabChip kit (Agilent Technologies, Madrid, Spain) was used. Only samples with RNA integrity number ≥ 9 were selected, as previously described [17].

### 2.5. Microarray Procedure

The differential expression profiling study was conducted on six samples for each experimental condition (REF, P, G, and S) obtained at the end of the lactation period using two-step mRNA PCR amplification with random primers and poly-T, followed by library generation and sequencing on the Illumina HiSeq X-Ten platform (Seqplexing Multiplex SL, Valencia, Spain). Briefly, the protocol involved amplifying the mRNA fragments using random and polyT primers and incorporated a unique molecular identifier (UMIs) to distinguish duplicates and ensure accurate fragment quantification. Library quality was assessed by QIAxcel Advanced System to detect degradation or low concentration prior to sequencing. Sequencing was performed on the Illumina HiSeq X-Ten with paired-end 2 × 150 bp reads, although reverse reads (R2) were excluded due to low quality caused by poly-A tails. Raw FASTQ data were used for bioinformatic analysis.

### 2.6. Microarray Data Analysis

The bioinformatics pipeline began with quality control and trimming of raw sequencing reads, removing adapters and poly-A sequences. FastQC evaluated the quality of FASTQ files, ensuring good %Q20 scores and absence of errors. UMIs were processed using UMI-tools, relocating molecular markers to the read header for accurate mapping. Reads were aligned to the “mRatBN7.2” reference genome with STAR, accounting for splicing junctions. Duplicate reads were removed using UMIs to distinguish unique molecules. Gene expression was quantified using HTSeq-count and normalized to adjust for sequencing depth. Differential expression analysis was performed with DESeq2, identifying significant gene differences. PCA clustering in R evaluated sample quality, while visualization tools like ggplot and pheatmap facilitated data interpretation.

The expression of each gene was reported as the base 2 logarithm of ratio of the value obtained of each condition relative to the control condition (REF group). The cutoff values for log2fold change (log2FC) were set at 1 for upregulated and −1 for downregulated genes. A gene was considered differentially expressed if it displayed a PFP (percentage of false prediction, equivalent of false discovery rate, FDR) less than 0.05 by rank product non-parametric method (RankProd).

Venn Diagrams in GX allowed identification of differently expressed genes that follow the same pattern (e.g., upregulated or downregulated) among the experimental conditions. Finally, up- and downregulated genes were analyzed in terms of gene ontology using a hypergeometric analysis (GOStats). The output of this analysis was then filtered using two different criteria. On the one hand, data was filtered by statistical significance (adjusted *p*-value) and, on the other hand, data was ordered by fold expression.

### 2.7. Validation of Gene Expression by Real Time PCR

Two micrograms of total RNA were transcribed into cDNA. Selected targets were measured using specific TaqMan^®^ primers and probes for PCR (Applied Biosystems, AB, Weiterstadt, Germany): *Fam117a* (Rn01304319_m1), *Ets1* (Rn01524757_m1), *Hmox1* (Rn00561387_m1), *Apln* (Rn00581093_m1), *Il17* (Rn01757168_m1), *Fasn* (Rn00569117_m1), and *Lsd1* (Rn01181029_m1).

Quantitative Real-Time PCR assays were performed in duplicate for each sample using the ABI PRISM 7900HT Sequence Detection System (Applied Biosystems). Gene expression levels were normalized to the housekeeping gene *Gusb* (Rn00566655_m1). Data analysis was conducted using SDS v2.4 software (Applied Biosystems). Results are presented as fold changes in target mRNA expression relative to the endogenous control, calculated using the standard 2^−ΔΔ^Ct method. Comparisons were made across the different experimental groups, with values from the REF group serving as the baseline, representing a one-fold change in gene expression [17].

### 2.8. Ferritin and Adipsin Quantification

Plasma ferritin levels were quantified using an ELISA kit (Invitrogen, Carlsbad, CA, USA) according to the manufacturer’s protocol. Briefly, plasma samples and standard dilutions were added to the wells of a pre-coated plate and incubated for 90 min at 37 °C. Following incubation, the appropriately diluted biotinylated detection antibody was added, followed by incubation for 60 min at 37 °C. After washing the plate thoroughly, avidin-horseradish peroxidase (HRP) solution was added to each well and incubated for 30 min at 37 °C. The plates were then washed, and the substrate reagent was added for 15 min at 37 °C. The reaction was terminated by the addition of stop solution. Optical density (OD) was measured at 450 nm using a microplate photometer (Labsystems Multiskan, Helsinki, Finland). Data were interpolated using standard curves generated using Ascent software v2.6 (Thermo Fisher Scientific, S.I.U., Barcelona, Spain) and expressed as ng/mL.

Plasma adipsin levels were measured using the Rat Complement Factor D/Adipsin ELISA kit (Biotechne, Minessota, MI, USA) following the manufacturer’s instructions. Briefly, appropriate diluted samples and standard dilutions were added to the pre-coated plate and incubated for 90 min at 37 °C. Then, the biotinylated detection antibody was added to the plate and incubated for 60 min at 37 °C. After washing, avidin-HRP conjugate was added to each well and incubated for 30 min at 37 °C. The plates were then washed, and the substrate reagent was added to the plates and incubated for 15 min at 37 °C. The enzyme-substrate reaction was terminated by the addition of stop solution. OD at 450 nm by a microplate photometer as previously described.

### 2.9. Real Time PCR and ELISA Statistics

Results from Real Time PCR and ELISA were statistically analyzed using the SPSS 22.0 software package (SPSS, Inc., Chicago, IL, USA). Levene’s test and the Kolmogorov–Smirnov test were used to evaluate homogeneity of variances and normal distribution, respectively. Nonparametric tests were conducted when the assumptions of normality and variance equality were not met. Specifically, the Kruskal–Wallis test and the Mann–Whitney U test were employed to determine significance for independent samples. A significance level of adjusted *p* < 0.05 was considered statistically significant.

## 3. Results

### 3.1. Effect on Overall Intestinal Rat Gene Expression

**Figure 1:**
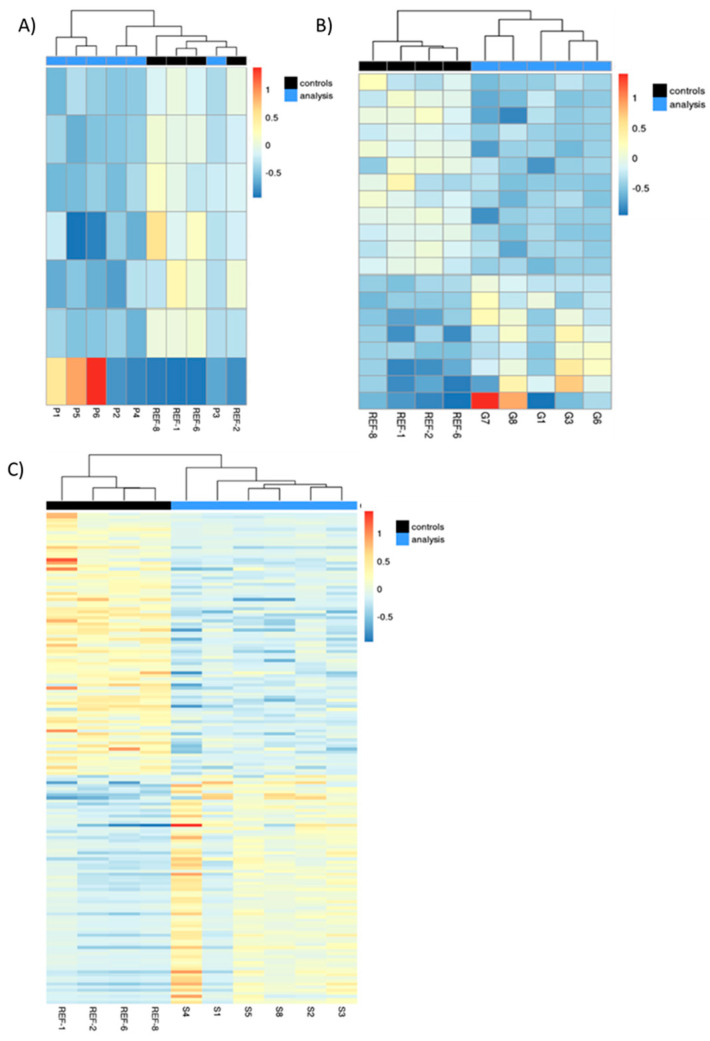
Heatmap of the genes significantly modified by the HFP diet, either: (**A**) during the three weeks prior to gestation (pre-gestation or P group), (**B**) during the three weeks of gestation (gestation or G group), or (**C**) during the three weeks of lactation (S group). The color scale indicates the log 2-fold change for all three panels. In all cases, REF animals are displayed in black (controls), and animals receiving the HFP diet are shown in blue (analysis: nutritional intervention) (*n* = 4–6/group). Grouping of animals based on similarity is displayed at the top of each panel.

In the small intestine, the gene expression was affected by the nutritional intervention, with different intensity depending on the period at which the diet was administered. The number of genes with a significant log2 fold change were 7, 20, and 114 in the P, G, and S groups, respectively. The S group, which received the HFP diet during lactation, i.e., closer to the sampling day (end of lactation), showed the highest number of changes (Figure 1). In all cases, up- and downregulated genes were distributed in similar proportions (40–60%).

Most of the modified genes were associated with immune signaling, intestinal absorption, and cell growth (differentiation and structure development), as determined by gene ontology (GO) biological processes (BP). In addition, the majority of the genes significantly modulated in the three groups were involved in Molecular Functions (MF) such as DNA and RNA binding. Genes modulated in the G and S groups were also associated with MF activities, including hydrolase, catalytic, transporter, or molecular transducer activities.

**Figure 2:**
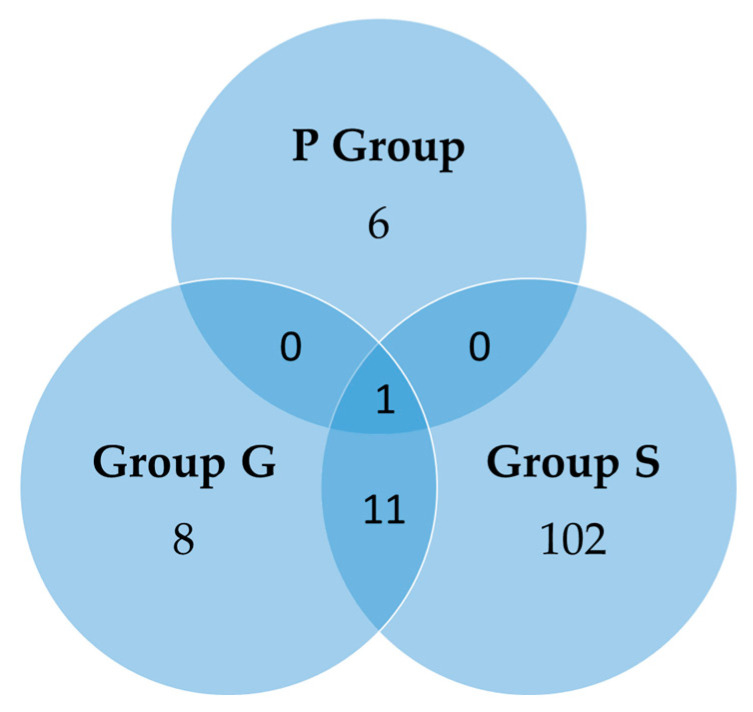
Venn diagram displaying statistically significant differentially up- and downregulated genes in pre-gestation group (P group), gestation group (G group), and lactation group (S group) with respect to the REF group (*n* = 4–6/group).

In addition, very few genes influenced by the diet were shared among groups (Figure 2). Only one gene was affected by the HFP diet independently of the period in which the diet was given; 12 up- or downregulated genes differently expressed with respect to the REF group were shared between G and S groups.

### 3.2. Gene Expression Changes Due to the HFP Diet During Pre-Gestation

The HFP diet administered to rats during the three weeks prior to gestation (P group) induced the regulation of a small number of genes at the end of lactation (Table 2).

In the P group, the HFP diet significantly downregulated *Foxq1*, a member of the FOX gene family involved in embryonic development, cell cycle regulation, tissue-specific gene expression, cell signaling, and tumorigenesis [18]. Moreover, downregulation of the sodium-dependent glucose transporter 1A (*Mfsd4b1*) and *Fam117a*, which is a C/EBP-induced protein with mostly unknown functions [19] was detected. Only one gene was found overexpressed: *Mptx1* (a mucosal pentraxin pseudogene predicted to enable metal ion binding activity).

It is noteworthy that *Fam117a* is the only gene significantly modified, with a lower Log2Fc, in all three periods of intervention: pre-gestation, gestation, and lactation.

### 3.3. Gene Expression Changes Due to the HFP Diet During Gestation

The animals that received the HFP diet during the three weeks of gestation (G group) showed a higher number of genes regulated with respect to the previous period (Table 3).

Among the downregulated genes, displayed according to significance (Table 3A), *Arid1a* (AT-rich interaction domain 1A), which encodes a protein that is part of a large ATP-dependent chromatin remodeling complex, was the gene most significantly affected by the HFP diet during gestation (*p* < 10 × 10^6^). In addition, *Ppp1r26* (predicted to enable protein phosphatase inhibitor activity) and *Nudt21* (a protein involved in 3′ RNA cleavage and polyadenylation processing), were also downregulated. For the upregulated genes (Table 3B), several were involved in lipidic metabolism, such as the *Fasn* (fatty acid synthase), *Lpl* (lipoprotein lipase), and *Creb314* (associated with adiposity). Other genes were linked to immune response, such as the *Cfd* (complement factor D), *Spon-2* (enabling cell adhesion and migration), and *Hmox1* (participating in heme group degradation).

*Fam17A* gene can also be found in Table 3A, indicating that this gene may play a role in the effects induced by fiber and polyphenols not only prior to gestation, as mentioned above, but also during gestation, with effects lasting until the end of the lactation period.

### 3.4. Gene Expression Changes Due to the HFP Diet During Lactation

Rats fed the HFP diet during the three weeks of lactation (S group) showed the highest number of modified genes compared to those fed the REF diet (Table 4).

Three of the significantly downregulated genes in the G group (*Arid1a*, *Ppr126,* and *Nudt21*) were also found to be downregulated in the S group, showing lower gene expression than in the REF group, but with higher significance (*p* < 10 × 10^−10^). In addition, *Fam117a*, a gene significantly downregulated in both the P and G groups, was also modified here with a very high level of significance.

Regarding the upregulated genes, *Sync* and *Fasn*, which encode syncoilin and fatty acid synthase, respectively, were significantly affected. However, other genes, such as *Cnih3,* which regulates the trafficking and gating properties of AMPA-selective glutamate receptors, showed even higher levels of upregulation.

### 3.5. PCR Confirmation of Key Genes

**Figure 3:**
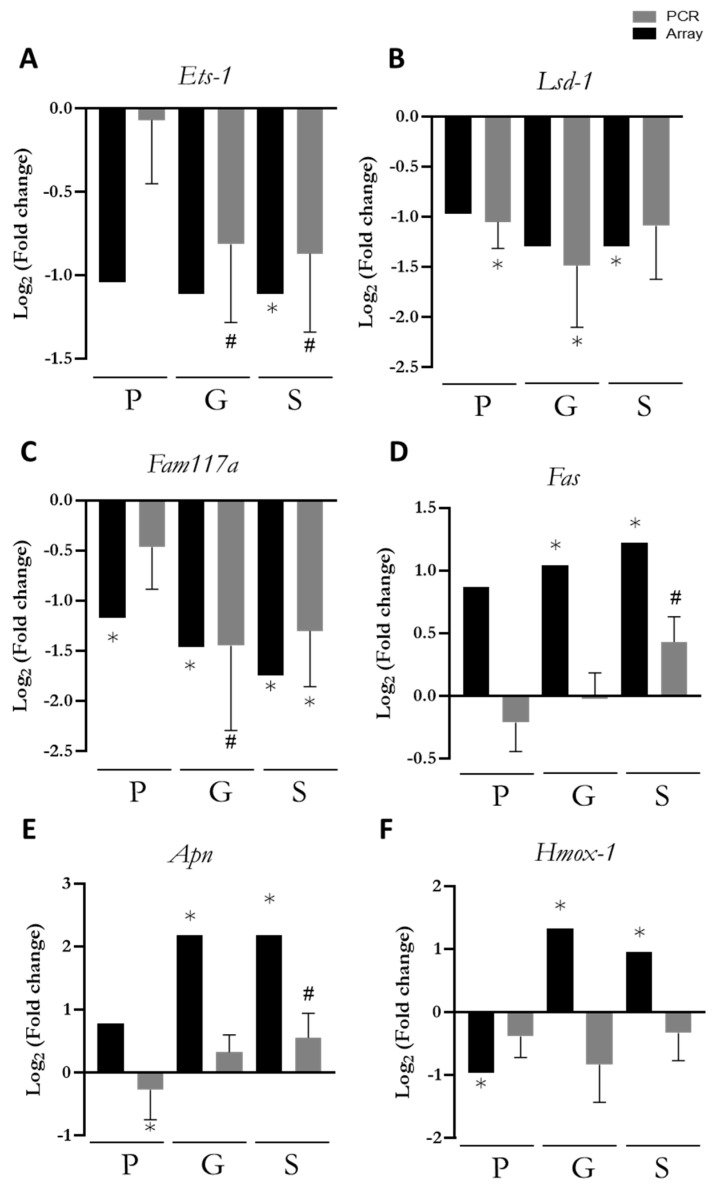
Comparison of fold change in (**A**) *Ets-1*, (**B**) *Lsd1*, (**C**) *Fam117a*, (**D**) *Fasn*, (**E**) *Apln,* and (**F**) *Hmox1* with respect to REF between the array results (black bars) and the PCR (grey bars) (*n* = 6), pre-gestation group (P group), gestation group (G group), and lactation group (S group). Statistical differences: * *p* < 0.05 vs. REF, # *p* < 0.1.

To confirm the array results, a PCR of key genes was performed (Figure 3). The selected genes were chosen for their specific roles and differential expression patterns in the array results. *Ets-1* (Protein *C-ets-1*), *Lsd1* (Lysine-specific demethylase), and *Fam117a* (Family with sequence similarity 117, member A), were included due to their consistent downregulation across the P, G, and S groups. *Fasn* (Fatty acid synthase) was chosen for its gradual upregulation, which varied depending on the nutritional intervention period.

Additionally, *Fasn*, *Hmox1*, and *Apln* (*Apelin*) were selected for their involvement in immune response, oxidative stress regulation, and lipid metabolism, which are all relevant to this study.

*Ets-1*, *Lsd1*, and *Fam117a* were consistently downregulated in all groups compared to the REF group (Figure 3A–C). The PCR validation (grey bars) confirmed some of the array results, such as the downregulation of *Fam117a* in the G and S groups. Overall, the PCR results showed expression patterns similar to those of the array.

*Fasn* and *Apln* (Figure 3D,E) PCR results showed that *Fasn* expression tended to increase only in the S group, while *Apln* was downregulated in the P group and upregulated in the S group. Lastly, the PCR results did not confirm the changes in *Hmox1* (Figure 3F) expression in the P, G, and S groups.

### 3.6. ELISA Confirmation of Upregulated Changes

**Figure 4:**
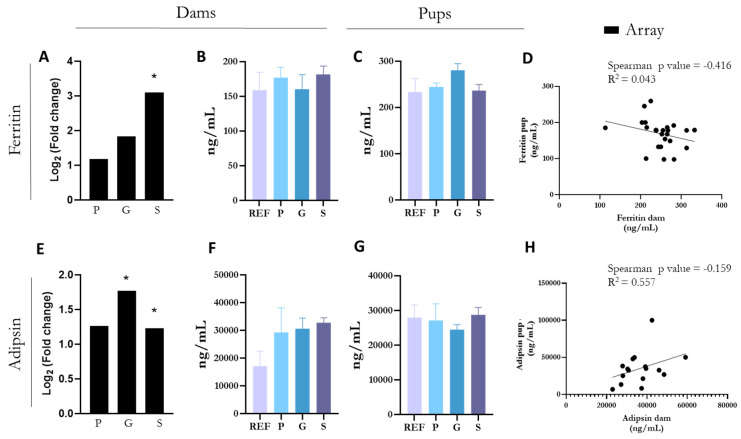
(**A**) Ferritin gene fold-change with respect to REF by array results. Ferritin plasma levels in (**B**) dams and (**C**) pups at the end of suckling by ELISA. (**D**) Correlation between plasma levels of ferritin in the mothers and in the offspring. (**E**) Adipsin gene fold-change with respect to REF by array results. Adipsin plasma levels in (**F**) dams and (**G**) pups at the end of suckling by ELISA. (**H**) Correlation between plasma levels of adipsin in the mothers and in the offspring. Data (**B**,**C**,**F**,**G**) are expressed as mean ± S.E.M. (*n* = 4–6 animals per group). Statistical differences: * *p* adj < 0.05 vs. REF.

To confirm the array results, the quantification of some proteins derived from key genes were performed in plasma (Figure 4). Ferritin and adipsin proteins were selected for analysis due to their significant changes observed in the array results. Ferritin gene exhibited a clear fold change increase in the small intestine of the S group after the nutritional intervention (Figure 4A), whereas adipsin gene levels were significantly elevated in both the G and S groups (Figure 4E). However, despite these changes in gene expression in the small intestine, protein validation in maternal plasma samples did not show significant differences between the groups (Figure 4B,F).

Furthermore, the levels of ferritin and adipsin were also measured in the plasma of pups at the end of the suckling period to assess whether the changes induced in the mothers were also induced in the offspring. The results indicated that the maternal diet did not affect the levels of ferritin (Figure 4C) or adipsin (Figure 4G) in the offspring. Additionally, a negative correlation was found between maternal and offspring ferritin levels (R^2^ = 0.043) (Figure 4D), whereas no significant correlation was observed between maternal and offspring adipsin levels (Figure 4H). All these results suggest local effects without systemic impact.

## 4. Discussion

Different factors affecting the mother, such as environmental conditions, nutrition, and health status, play an important role in fetal development, a process referred to as fetal programming [10]. An imbalanced maternal diet, such as a low-nutrient diet, can cause slowed growth and low birth weight, whereas an unhealthy diet may predispose children to overweight and obesity. Both situations, among others, can impact the fetus and increase the risk of illnesses later in life. In addition, emerging translational evidence suggests that epigenetic alterations (e.g., DNA methylation, miRNA expression, and histone modifications) may occur due to maternal diet and contribute to the risk of diseases later in life, such as diabetes, cardiovascular diseases, cancer, and neurological disorders [8,9]. In addition to the pre-gestational and gestational periods, lactation plays a crucial role in infant development, as breast milk provides numerous bioactive components extending beyond just nutrients [20]. Polyphenols and fiber, which are part of a healthy diet recommended for pregnant and lactating mothers, may contribute to these processes. Therefore, understanding the precise impact of dietary interventions, as well as the differential effects depending on the timing of these interventions, is of great importance.

The present study evaluated, at a pre-clinical level, the impact of a high-fiber and polyphenol (HFP) diet during pre-gestation, gestation, or lactation on the intestinal gene expression, where direct interaction between dietary compounds and the host occurs. The HFP diet demonstrated an influence on the mRNA levels of many genes involved in various biological processes (e.g., immune response, lipid metabolism, growth, etc.). Furthermore, some effects were consistent across the studied periods, such as the impact on the expression of *Fam117a* (Family with sequence similarity 117, member A).

The *Fam117a* gene has been identified as a critical regulator of gene expression, particularly in processes involving cellular proliferation, immune modulation, and tumor suppression. Its expression is associated with the regulation of pathways that influence cell cycle progression and immune cell infiltration, indicating its involvement in maintaining cellular homeostasis [19,21]. However, evidence regarding the function of *Fam117a* and its impact on health remains scarce, limiting our understanding of its broader biological roles. In the context of the small intestine, downregulation of *Fam117a* was observed in mothers fed a diet rich in fiber and polyphenols during any of the three periods studied, which may indicate a shift in the transcriptional landscape influenced by epigenetic mechanisms. This subexpression could reflect a diet-induced adaptation aimed at balancing intestinal immune responses or reducing proliferation-related pathways, aligning with the anti-inflammatory and antioxidant effects of fiber and polyphenols.

With regard to epigenetics, the findings of our study highlight a significant downregulation of the *Lsd1* (lysine-specific demethylase 1) gene in the small intestine of mothers fed a fiber- and polyphenol-rich diet during gestation (G group) and lactation (S group). Previous research highlights the critical role of *Lsd1* in maintaining intestinal homeostasis, regulating stem cell differentiation, and responding to environmental stimuli [12,22,23,24]. Furthermore, the interaction of dietary fiber and polyphenols with gut microbiota may contribute to the production of SCFAs, which are known to regulate epigenetic enzymes such as *Lsd1* [23]. In this context, the reduction in *Lsd1* expression could reflect an adaptive mechanism, potentially reprogramming the intestinal epithelium towards a reparative or anti-inflammatory state, as suggested in studies showing enhanced regenerative capacity in *Lsd1*-deficient models [24]. These findings emphasize the critical role of maternal diet in shaping gene expression patterns in the intestinal epithelium, with implications for gut homeostasis and immunity modulation during key developmental periods.

In addition to changes related to epigenetic modulation, the HFP diet also impacted the gene expression associated with growth and antioxidant status. For instance, the *Hmox-1 (Heme Oxygenase-1*) gene encodes an enzyme with a critical role in cellular defense against oxidative stress and inflammation. *Hmox-1* regulates gene expression by modulating oxidative pathways, influencing transcription factors, and promoting the expression of antioxidant and cytoprotective genes [25,26,27,28]. Overexpression of *Hmox-1* in the small intestine of mothers fed an HFP diet during gestation or lactation may indicate an enhanced protective response triggered by these dietary components. Fiber-derived metabolites, such as SCFAs, and polyphenols are known to activate *Hmox-1* expression, thereby reducing oxidative stress, enhancing intestinal barrier integrity, and mitigating inflammation. Although this overexpression was not confirmed in the PCR approach, it likely reflects an adaptive mechanism that enhances intestinal health and resilience, potentially benefiting both the mother and her offspring. Further investigation is warranted to explore how these dietary-induced changes in *Hmox-1* expression influence long-term intestinal function and overall health outcomes.

Our study reveals that a maternal diet enriched with fiber and polyphenols significantly upregulated the expression of the *Apln* gene in the small intestine during gestation and lactation. This finding aligns with the established role of the *Apln* signaling system in immune development, inflammation, vascular function, and metabolic homeostasis [29,30,31,32,33]. The increased expression of this molecule in response to maternal dietary intake may represent an adaptive mechanism to enhance intestinal repair, mitigate inflammation, and strengthen gut barrier integrity and immunity during this critical development period.

In line with this observation, the gene expression of ferritin, an iron-storage protein and key biomarker for iron levels [34], was significantly upregulated in response to the dietary intervention across the P, G, and S groups. A gradual increase in intestinal ferritin levels due to supplementation was also observed, with the highest levels seen in the S group. However, despite the observed changes in intestinal ferritin gene expression, ELISA validation revealed no differences in serum ferritin levels among the groups. This suggests that dietary intervention selectively influenced intestinal iron storage without systemic changes. Polyphenols, known for their antioxidant properties, have been reported to inhibit iron absorption by forming complexes with iron, potentially limiting its bioavailability. On the contrary, fiber and the acidification of the intestinal milieu through SCFAs production may increase iron absorption [35]. Furthermore, ferritin transfer during pregnancy and lactation remains poorly understood [36] and no significant changes in offspring ferritin levels were observed following maternal dietary intervention. Overall, the local effect of the HFP diet on intestinal ferritin expression deserves further study in order to understand its broader implications.

Maternal immunity regulation during gestation and lactation is critical. Whereas a predominant T helper (Th) 2 or anti-inflammatory response is required during gestation, a shift toward a Th1 or pro-inflammatory response occurs after birth [37]. These immune changes seem to be modulated by diet, and in this sense, our study observed changes in certain genes linked to immune response, as is the case of *Cfd* (complement factor D), *Spon-2* (enabling cell adhesion and migration), or more clearly, *Ets-1*, which was specially downregulated in mothers receiving the HFP diet during lactation. *Ets-1* is a critical regulator of immune cell functions, specifically contributing to cell proliferation, differentiation, and metastasis. Its expression in mice is prominently active during early postnatal development and becomes more restricted in adulthood [38]. Dysregulation of *Ets-1* has been linked to autoimmune disorders and cancer [39]. Epigenetic modifications play a significant role in regulating *Ets-1* expression, particularly in cancer [40], and polyphenols have been identified as potent epigenetic modulators [41]. In this context, the observed downregulation of *Ets-1* in the current study could potentially reduce the risk of health complications, including autoimmune and oncological diseases. These findings underscore the importance of dietary interventions, particularly those involving epigenetically active compounds, in modulating gene expression to support long-term immune health.

Finally, changes in genes involved in lipid metabolism and fat storage regulation have been also observed, particularly during intervention periods closer to the day of analysis, such as gestation and lactation (e.g., *Fasn, Lpl, Creb314,* and *adipsin*).

*Fasn* plays an essential role in *de novo* lipogenesis. Although previous studies have primarily focused on its role in various malignancies, where most nutritional interventions have been conducted, recent findings indicate that it also regulates the survival, differentiation, and function of immune cells [42,43]. Specifically, upregulation of *Fasn* expression promotes macrophage polarization from M0 to M1 and enhances their pro-inflammatory activity [42,43]. Moreover, it has been identified as a crucial metabolic regulator that drives the inflammatory subgroup of Th17 cells [44], while simultaneously contributing to the functional maturation of Treg cells [45]. In the present study, the microarray results, but not the PCR analysis, revealed that the intake of a HFP diet during gestation (G group) and lactation (S group) led to upregulated intestinal *Fasn* gene expression in dams. Further studies are needed to confirm these findings, elucidate the underlying mechanisms, and assess the potential implications for immune function and metabolic regulation under both physiological and pathological conditions.

On the other hand, adipsin, also referred to as complement factor D, was the most affected adipokine gene in the maternal small intestine in response to the dietary intervention, with increased expression observed in the G and S groups compared to the REF group. Adipsin, secreted predominantly by adipose tissue, plays a critical role in lipid metabolism and immune response linked to complement activation, and elevated plasma levels are commonly associated with obesity [46]. Recent studies have highlighted its importance during pregnancy, particularly in cases of maternal obesity, where placental macrophages secrete higher levels of adipsin, potentially leading to increased fetal adipsin levels [47]. Despite these findings, the impact of maternal diet on adipsin levels remains unclear. In our study, ELISA validation revealed no significant differences in plasma adipsin levels across groups, and a lack of changes or correlations were detected in offspring plasma adipsin levels following maternal intervention. Overall, the intestinal rise in adipsin gene expression could suggest a specific role for the complement pathway in these periods of development, while also potentially contributing to the local regulation of energy balance.

The present study has some experimental limitations besides the discrepancies between the array and the PCR results. The microarray outcomes should be interpreted as preliminary data, representing a first exploratory approach that can guide more targeted studies in the future. Thus, focusing on the pathways and specific genes affected by dietary intervention would be of interest. In addition, the impact of this particular diet does not allow distinguishing between the effects derived from the polyphenols and the fiber metabolism, and future approaches could include separate dietary interventions to determine whether the observed effects are attributed to a single component or the result of a synergistic relationship between fiber and polyphenols.

## 5. Conclusions

Overall, the key significance of the present study lies in evidencing that maternal diet has an influence on intestinal gene expression during critical offspring development periods. In addition, the changes are less pronounced when the dietary intervention occurs further from the analysis time, highlighting the short-lived effects on some genes. *Fam117a* arises as a key gene involved in the impact and long-lasting effect of a diet rich in fiber and polyphenols across all the three periods studied: pre-gestation, gestation, and lactation. Its regulation could suggest a potential role in orchestrating transcriptional and epigenetic adaptations that influence cellular and immune dynamics, highlighting its importance in shaping long-term health outcomes associated with maternal nutrition.

## Figures and Tables

**Table 1:** Composition of the experimental diets used in the study.

Components	REF Diet (g/kg)	HFP Diet (g/kg)
Casein	200	200
L-Cysteine	3	3
Cornstarch Flour	379.186	289.186
Inulin	0	80
Pectin	0	10
Maltodextrin	132	132
Sucrose	100	100
Soybean Oil	70	70
Cellulose	50	50
Mineral Mix (TD94049)	48	48
Ferric Citrate	0.3	0.3
Vitamin Mix (TD94047)	15	15
Choline Bitartrate	2.5	2.5
Tertiary Butylhydroquinone	0.014	0.014
Polyphenols	0	5
Catechin	0	1
Epicatechin	0	1
Hesperidin	0	1.5
Naringenin	0	0.75
Quercetin	0	0.75

**Table 2:** List of the seven genes (**A**) downregulated and (**B**) upregulated in the small intestine of rats that received the high fiber and polyphenols diet (HFP) for the three weeks prior to conception, compared to REF group (*n* = 4–6/group). All gene changes in P group vs. REF group are ordered by adjusted *p* value (padj).

(A) Downregulated Genes			
Gene	Name	Log2FC	padj
ENSRNOG00000022136	*Loc685680*	−1.38	1 × 10^−3^
ENSRNOG00000024543	*Foxq1*	−1.39	3 × 10^−3^
ENSRNOG00000062314	*Mfsd4b1*	−1.05	3 × 10^−3^
ENSRNOG00000004417	*Fam117a*	−1.17	5 × 10^−3^
ENSRNOG00000003681	*Lct*	−1.20	2 × 10^−2^
ENSRNOG00000019813	*Ppp4*	−1.36	4 × 10^−2^
**(B) Upregulated genes**			
**Gene**	**Name**	**Log2FC**	**padj**
ENSRNOG00000046165	*Mptx1*	5.69	6 × 10^−3^

**Table 3:** List of the 20 genes (**A**) downregulated and (**B**) upregulated in the small intestine of rats that received the high fiber and polyphenols diet (HFP) during the three weeks of gestation compared to REF group (*n* = 4–6/group). All gene changes in the G group vs. REF group are ordered by adjusted *p* value (padj).

(A) Downregulated Genes			
Gene	Name	Log2FC	padj
ENSRNOG00000006137	*Arid1a*	−1.02	8 × 10^−6^
ENSRNOG00000027193	*Ppp1r26*	−1.07	2 × 10^−4^
ENSRNOG00000042983	*Nudt21*	−1.04	2 × 10^−4^
ENSRNOG00000027739	*Cndp1*	−1.16	2 × 10^−4^
ENSRNOG00000017332	*Dapk2*	−1.01	1 × 10^−3^
ENSRNOG00000011677	*Slc39a10*	−1.29	2 × 10^−3^
ENSRNOG00000042758	*Tmem243*	−1.03	6 × 10^−3^
ENSRNOG00000004417	*Fam117a*	−1.46	7 × 10^−3^
ENSRNOG00000021053	*Lsr*	−1.03	1 × 10^−2^
ENSRNOG00000006663	*Uch2*	−1.08	1 × 10^−2^
ENSRNOG00000008584	*Rnaseh1*	−1.17	3 × 10^−2^
ENSRNOG00000006600	*Unk*	−1.06	4 × 10^−2^
**(B) Upregulated genes**			
**Gene**	**Name**	**Log2FC**	**padj**
ENSRNOG00000033564	*Cfd*	1.77	1 × 10^−3^
ENSRNOG00000023493	*Creb314*	1.02	6 × 10^−3^
ENSRNOG00000006033	*Spon-2*	1.03	6 × 10^−3^
ENSRNOG00000045636	*Fasn*	1.04	8 × 10^−3^
ENSRNOG00000014117	*Hmox1*	1.32	8 × 10^−3^
ENSRNOG00000012181	*Lpl*	1.41	2 × 10^−2^
ENSRNOG00000068020	*Uqcc5*	1.01	3 × 10^−2^
ENSRNOG00000003984	*Apln*	2.18	2 × 10^−3^

**Table 4:** List of the top 10 (**A**) downregulated and top 10 (**B**) upregulated genes from the 114 significantly modified genes in the small intestine of rats receiving the high fiber and polyphenols diet (HFP) during the three weeks of lactation compared to REF group (*n* = 4–6/group). All gene changes in the S group vs. REF group are ordered by adjusted *p* value (padj).

(A) Downregulated Genes			
Gene	Name	Log2FC	padj
ENSRNOG00000006137	*Arid1a*	−1.05	1 × 10^−16^
ENSRNOG00000027193	*Ppp1r26*	−1.28	1 × 10^−11^
ENSRNOG00000021081	*Vps72*	−1.27	6 × 10^−11^
ENSRNOG00000019671	*Rsbn1*	−1.09	1 × 10^−10^
ENSRNOG00000042983	*Nudt21*	−1.26	2 × 10^−9^
ENSRNOG00000004417	*Fam117a*	−1.74	1 × 10^−8^
ENSRNOG00000042758	*Tmem243*	−1.21	2 × 10^−8^
ENSRNOG00000011677	*Slc39a10*	−1.29	4 × 10^−6^
ENSRNOG00000002032	*Ifngr2*	−1.02	1 × 10^−5^
ENSRNOG00000053599	*Pogz*	−1.00	1 × 10^−5^
**(B) Upregulated genes**			
**Gene**	**Name**	**Log2FC**	**padj**
ENSRNOG00000008118	Sync	1.31	2 × 10^9^
ENSRNOG00000045636	*Fasn*	1.22	5 × 10^9^
ENSRNOG00000017178	*Hydin*	5.91	1 × 10^−4^
ENSRNOG00000063281	*60S ribosomal protein L29*	5.78	4 × 10^−4^
ENSRNOG00000003984	*Apln*	2.18	2 × 10^−3^
ENSRNOG00000022724	*Cnih3*	5.26	3 × 10^−3^
ENSRNOG00000043103	*Frrs1l*	2.49	3 × 10^−3^
ENSRNOG00000003741	*Nptx1*	1.97	5 × 10^−3^
ENSRNOG00000033564	*Cfd*	1.22	5 × 10^−3^
ENSRNOG00000003785	*Usp43*	1.18	6 × 10^−3^

## Data Availability

The datasets generated and/or analyzed during the current study are available from the corresponding author on reasonable request. The data are not publicly available due to ongoing further research.
